# Impact of data source and time reference of functional status on hospital mortality prediction

**DOI:** 10.1186/1472-6963-12-115

**Published:** 2012-05-14

**Authors:** Woan Shin Tan, Yew Yoong Ding, Wai Fung Chong, Jam Chin Tay, Jackie Yu-Ling Tan

**Affiliations:** 1 Health Services and Outcomes Research Department, National Healthcare Group, Singapore, Singapore; 2 Department of Geriatric Medicine, Tan Tock Seng Hospital, Singapore, Singapore; 3 Department of General Medicine, Tan Tock Seng Hospital, Singapore, Singapore

## Abstract

**Background:**

The study objective was to compare physical function documented in the medical records with interview data, and also to evaluate hospital mortality predictions using pre-admission and on-admission functional status derived from these two data sources.

**Methods:**

A prospective cohort study of 1402 subjects aged 65 years and older to the general medicine department of an acute care hospital was conducted. Patient-reported pre-admission and on-admission functional status for impairment in any of the five activities of daily living (ADLs) items (feeding, dressing, grooming, toileting and bathing), transferring and walking, were compared with those extracted from the medical records. For the purpose of mortality prediction, pre-admission and on-admission impairment in transferring from the two data sources were included in separate multivariable logistic regression models. We used a variable selection method that combines bootstrap resampling with stepwise backward elimination.

**Results:**

For all ADL categories, the agreement between the data sources was good for pre-admission functional status (k: 0.53–0.75) but poor for on-admission status (k: 0.18–0.31). On-admission impairment was higher in the medical records than at interview for all basic ADLs. Using interview data as the gold standard, although sensitivity for pre- and on-admission ADLs was high (59–93%), specificity for on-admission status was poor (30–37%). The pre-admission models using interview data predicted mortality better than the model using medical records (c-statistic: 0.83 versus 0.82). Similar results were found for models incorporating on-admission functional status (c-statistic: 0.84 versus 0.81). However, the differences between the four models were not statistically significant.

**Conclusion:**

Medical records can be a good source for pre-admission functional status but on-admission functional impairment was over-reported in the medical records. The discriminatory power of the hospital mortality prediction model was significantly improved with the incorporation of functional status information but it was not significantly affected by their time reference or source of data.

## Background

Functional status has been found in many studies to be a key predictor of hospital mortality for older patients [1-3]. Reflecting the impact of illness severity on the whole person and on the individual's ability to negotiate the external environment, it may contain more important information about prognosis than admitting diagnosis, comorbid illness burden or routine physiologic measures [4-6]. Functional status is determined by the ability to perform activities of daily living (ADL). Measured prior to hospital admission, it is a sign of past health status and can indicate risk of further functional decline. During acute illness, the elderly are at higher risk of impairment in function. On admission to the hospital, functional status reflects the impact of recent illness on the patient superimposed on pre-admission functioning.

While recent studies [7-10] have examined the impact of functional trajectories on post-discharge mortality of between 3 and 6 months, there is little consensus in literature regarding whether a static or time-dependent measure should be used for mortality prediction. While functional status at the point of hospitalization [4,7,11] is a widely used static measure, it has been found to be a weaker predictor of 1-year mortality in acutely ill patients than functional status before the onset of acute illness [12]. For shorter term mortality such as inpatient death, there is little information in literature regarding the appropriate time reference.

To derive functional status information, studies have relied on either prospective interviewing of patients or documentation in medical records. However, different data sources can have possible biasing effects. Patient-reported outcomes have been found to better reflect observed function than physician report [6,13,14]. Likewise, compared with data derived from interviews, medical records have been reported to be a poor source for functional status measures [6,15]. This dissonance could be a result of differences in the purpose and method of data collection and the treatment of the marginally impaired patient.

Studies have highlighted the importance of gathering functional status data to facilitate its use as a prognostic or risk-adjustment variable. However, few studies [15] have assessed the relationship between data source and data quality, and its ensuing impact on the prediction of health-related events. Since timing and sources may have a joint impact on the quality of functional status information, it will be insightful to examine this. Therefore, the goal of this study was to compare medical record documentation against information derived from a prospective interview pertaining to physical function prior to, and at the time of hospitalization. In addition, we will investigate the joint impact of data sources and timing of functional status measurement on hospital mortality prediction.

## Methods

### Study participants

The study was carried out at the Tan Tock Seng Hospital. The 1200-bedded hospital is a public sector facility with 390 general medicine beds. It is the second largest acute care general hospital in Singapore. Subjects aged 65 years and older, admitted consecutively on weekdays between 6^th^ April and 29^th^ October 2008 to the general medicine department were included. There were 2,171 potential subjects. Patients were excluded if they could not be interviewed for reasons including intubation, coma, severe aphasia, or terminal condition (n = 326); if they were discharged or transferred or died within 48-hours (n = 53); if they declined participation (n = 90); or others (patient was asleep, not in the ward) (n = 282). Medical notes for 18 subjects were not available. The final sample included 1402 participants. Informed consent was obtained from all participants.

The study was approved by the Institutional Review Board of the National Healthcare Group, Singapore.

### Data collection

Trained research assistants carried out structured interviews with the patients or their proxies within 48 hours of admission. The interview included information pertaining to socio-demographics, level of social support, pre-admission and on-admission basic ADLs. Data on primary diagnosis and hospital mortality were extracted from the hospital’s administrative database.

Another research assistant, blinded to the study aims and the results of the patient interviews, abstracted data on co-morbid conditions and pre-admission and on-admission functional status of patients from the medical records. The nurse-trained abstractor reviewed the medical record, including notes of medical and nursing staff.

### Study variables

Based on clinical judgment and predictors found to significantly predict mortality in older patients in literature [1,16-19], we collected the following variables: demographics (age, sex, ethnic group), marital status, housing type, primary diagnosis, co-morbid disease burden and functional status.

The baseline interview included questions regarding basic ADLs. Patients or their proxies were asked if they experienced difficulty in the performance of each of the ADLs (feeding, bathing, grooming, dressing, toileting, transferring and walking) 2-weeks prior to being hospitalized, and at the point of admission. All items were rated either yes or no. Patients who used an adaptive device for an ADL function were not considered impaired if assistance from another person was not required.

Pre-admission function is routinely assessed by doctors as part of history taking and documented in the medical records. There are three checkboxes allowing doctors to indicate whether the patient is “independent”, “needs assistance” or “totally dependent” for the different functional aspects. Impairment in feeding, bathing, grooming, dressing, or toileting was captured as a whole whereas walking was a separate category. Information on transferring was obtained from documentation by doctors or therapists. On the other hand, assessment and documentation of on-admission function was done by ward nurses regarding patients’ level of nursing care needs. The checklist used asked whether patients require assistance in the following ADL categories: feeding, dressing or grooming, toileting or bathing, transferring, and walking. All items were rated either yes or no in the medical records.

Due to the differences in data collection system in the interview and medical record, we compared the difficulty experienced by the patient in performing at least 1 of 5 basic care skills (feeding, bathing, grooming, dressing, toileting), transferring, and walking. We also examined impairment in at least 1 of these 7 ADLs.

There were a total of 165 different primary diagnoses in the study sample. Only primary diagnoses, which are highly predictive of mortality in older hospitalized patients, were coded. This was based on the High-Risk Diagnoses for the Elderly Scale [18], which comprises 22 diagnostic groups. They include: Bone marrow failure, cancer (metastatic), cancer (solid tumour, localised), cirrhosis/end-stage liver disease, congestive heart failure, decubitus ulcer, delirium, dementia, lymphoma/leukaemia, major depression, malnutrition, renal failure (acute), renal failure (chronic), respiratory failure, chronic obstructive pulmonary disease, diabetes mellitus with end-organ damage, major stroke, multiple trauma, myocardial infarction, pneumonia and severe peripheral vascular disease. In our study, the International Classification of Disease, ninth version was used to identify, and group the diagnoses. Diagnoses with a prevalence of less than 1% in the sample were excluded from further analysis to avoid having too few observations per cell for analysis [20].

The Charlson Comorbidity Index (CCI) is a weighted prognostic index based on the number and severity of 19 comorbid conditions [21]. It was computed by the research team for the purpose of this study based on medical history in the medical records and diagnoses listed at admission. The medical conditions are weighted 1–6 with total scores ranging from 0–37. It was developed originally to predict 1-year mortality of internal medicine patients. Data abstracted from the medical record were used to compute the score. We have coded the score into four categories 0, 1, 2 and ≥ 3.

### Statistical analysis

#### Comparison of data sources

Pre-admission and on-admission functional status obtained from the interview was compared with results extracted from the medical notes. Cohen's kappa coefficient κ was computed to measure the level of agreement between the sources of data. Excellent agreement beyond chance is indicated by κ values above 0.75, 0.40 through 0.75 represent fair to good agreement and values below 0.40 indicate poor agreement [22]. Using data from the patient or proxy interviews as the reference standard [23], we computed the sensitivity, specificity, negative and positive agreement values. Since we cannot assume non-documentation to be equivalent to functional independence [6], we have excluded cases with missing documentation when comparing between data sources.

#### Development of hospital mortality prediction models

Katz *et al.* suggested that functional abilities are lost in the opposite order to which they were gained during childhood (first, feeding and transfer; later toileting and dressing; last bathing) [24]. Therefore, in the prediction of mortality, we have defined “ADL impairment” as needing physical assistance in transferring rather than as impairment in one of the seven basic care skills. Due to the hierarchy of impairment in basic ADLs, the latter measure would have categorised patients requiring assistance in bathing as functionally impaired, which may not sufficiently discriminate the survivors from those who died.

Bivariate analyses were conducted to examine the relationship between inpatient mortality and each variable including pre- and on-admission functional status drawn from the medical records and from the patient interviews. We used the Chi-square test or the Kruskal-Wallis test to assess differences among or between groups. Variables statistically significant at *P* < 0.25 were selected for possible inclusion in multivariable logistic regression models to predict hospital mortality. We chose *P* = 0.25 as the threshold for including variables in the multivariable model because this has been suggested elsewhere as an appropriate threshold [25].

The following four functional status variables were included in separate multivariable logistic regression models: “pre-admission ADL impairment” based on interview, “pre-admission ADL impairment” based on medical records, “on-admission ADL impairment” based on interview and “on-admission ADL impairment” based on medical records. For missing pre- and on-admission functional status data in the medical records, the median value adjusted for mortality and diagnosis, was used.

We adopted a model selection method that combines bootstrap resampling with automated variable selection methods [26]. Since the use of automated variable selection methods with logistic regression has been found to result in the identification of nonreproducible models, this approach allowed us to determine with greater confidence that a variable is indeed an independent predictor from its empirical distribution [27]. Our model selection method was based upon drawing 1000 repeated bootstrap samples (the Stata userwritten “swboot” command) from the original dataset. Within each bootstrap sample, backwards elimination was applied with removal criteria set at *P* < 0.10. For each variable, the proportion of bootstrap samples in which that variable was identified as an independent predictor of the outcome is determined. Variables that were significant in at least 60% of the bootstrap samples were included in the model [26]. Results were considered significant at *P* < 0.05.

The area under the receiver operating characteristic curve (AUC) or the c-statistic was used to evaluate the discriminatory power of the different predictive models [28]. Paired comparisons of the AUC based on different sources of data were conducted using the Hanley and McNeil method [29]. The Hosmer-Lemeshow test was used to examine the models’ goodness of fit [25]. The different models were ranked according to the Akaike's information criterion (AIC), with the one having the lowest AIC being the best. Analyses of the data were performed using STATA version 9.2 (Stata Corp, College Station, Texas).

## Results

### Comparison of data sources

Patient characteristics are shown in Table 1. The prevalence and agreement between interview and medical records data as well as sensitivity and specificity of basic ADL impairment in the medical records are can be found in Table 2. The overall prevalence of missing documentation ranged between 1.4% and 12.7%.

**Table 1:** Characteristics of study group (N = 1402)

**Characteristics**	**Study Group**
**Mean age, y (SD)**	77.8 (7.8)
**Female, %**	59.7
**Ethnic Group, %**	
Chinese	81.0
Malay	9.6
Indian	7.3
Others	2.1
**Marital status**	
Married	45.4
Widowed	50.4
Single	2.9
Divorced/Separated	1.4
Housing type*	
1-2 room public flat	8.9
3-5 room public flat	73.1
Private housing	11.9
Others (Sheltered housing)	6.0
**Primary Diagnosis ≥1% Prevalence, %**	
Pneumonia	16.6
Diabetes Mellitus with End Organ Damage	4.5
Congestive Heart Failure	2.3
Chronic Obstructive Pulmonary Disease	2.0
Renal Failure	1.9
Sepsis	1.8
Acute Myocardial Infarction	1.7
Cancer (solid tumor, localized)	1.2
Major Stroke (hemiplegia)	1.1
**Charlson Comorbidity Index, %**	
= 0	17.5
= 1	23.5
= 2	21.7
≥ 3	37.3
**Pre-admission, Any ADL Impairment, % †**	47.6
**On-admission, Any ADL Impairment, % †**	50.4
**Hospital mortality, %**	6.0

* Missing data were present for the following variables: Housing type (9 subjects).

† Based on interview with patients and their proxies.

S.D: standard deviation; ADL: Activities of Daily Living.

**Table 2:** Comparison of interview and medical record data for basic ADL impairments (N = 1402)

**Measure**	**Valid**	**Prevalence of Impairment (%)**						
		**Interview**	**Medical Records**	**κ**	Sensitivity* (%)	Specificity* (%)	PPV* (%)	NPV* (%)
**Pre-admission**								
Any of 5-item ADL†	1,383	33	38	0.71	87	87	77	93
Transferring	1,224	36	31	0.75	93	89	73	97
Walking	1,382	46	29	0.53	57	95	90	72
Any of 7-item ADL†	1,387	48	39	0.64	72	91	88	78
**On-admission**								
Any of 5-item ADL†	1,375	36	78	0.24	97	33	45	95
Transferring	1,376	27	78	0.18	98	30	34	98
Walking	1,375	49	78	0.31	94	37	59	88
Any of 7-item ADL†	1,374	50	83	0.28	97	31	59	91

* Interview data used as reference standard for sensitivity, specificity, positive predictive value (PPV) and negative predictive value (NPV).

† Includes feeding, bathing, grooming, dressing, and toileting.

ADL: Activities of Daily Living.

The medical records prevalence of pre-admission ADL impairment was higher for impairment in at least 1 of the 5-item ADL but lower for impairment in transferring and walking (Table 2). For impairment in at least 1 of the 7-item ADL, the medical records prevalence of pre-admission ADL impairment was within a 10% range of that recorded in the patient interview. For pre-admission functional status, the agreement between the data sources was good with kappa ranging between 0.53 and 0.75. Using interview data as the gold standard, sensitivity and specificity were high for pre-admission ADLs (72-93%) except for ambulation. Only 57% of patients who reported to require assistance in walking were reflected as such in the medical records.

For on-admission functional status, compared with interview data, the prevalence of impairment based on medical records data was consistently higher across all ADL categories. The agreement between the two data sources was poor with kappa ranging between 0.18 and 0.31 (Table 2). Although the sensitivity of medical records was high, specificity was consistently below 40%. The proportion of subjects with documented impairment in transferring who responded similarly during the interview was low (34%).

Regardless of time reference of the data, the agreement between patient interview and medical records was higher in proxy respondents than patient respondents (Additional file 1). The prevalence of impairment was also consistently higher in the medical records for both respondent groups.

### Hospital mortality prediction models

In the 1000 bootstrap samples, the number of times that each variable was identified as a significant predictor in each of the four iterations of the multivariate logistic regression model is summarised in Table 3. Overall, impairment in transferring, Charlson Co-morbidity Index scores (≥3); and primary diagnoses of pneumonia, acute myocardial infarction, sepsis, and renal failure were consistently selected in more than 60% of the samples across models. The results of the final regression models, which incorporated variables found to be significant in 60% of the bootstrap sample are shown in Table 4.

**Table 3:** Frequency with which each candidate variable was selected using backwards model selection in 1000 bootstrap samples

**Variable**	**Model Including Pre-admission ADL Impairment**		**Model Including On-admission ADL Impairment**	
	**Interview**	**Medical Record**	**Interview**	**Medical Record**
ADL impairment	1000	1000	1000	614
Pneumonia	996	994	969	999
AMI	945	961	971	963
Renal Failure	828	841	838	832
Sepsis	809	808	823	862
CCI ≥ 3	750	758	730	902
Chinese	570	637	611	642
Age	269	272	213	633
CCI = 0				
CCI = 1	188	201	232	247
CCI = 2	267	255	270	166
Sheltered home resident	251	208	147	110
Diabetes Mellitus with End Organ Damage	10	5	2	3

ADL: Activities of Daily Living; AMI: Acute Myocardial Infarction; CCI: Charlson Comorbidity Index.

**Table 4:** Multiple regression analysis predicting hospital mortality using interview and medical records data (N = 1402)

	**Model with Pre-admission ADL Impairment**		**Model with On-admission ADL Impairment**	
	**Interview**	**Medical records**	**Interview**	**Medical records**
	**OR (CI)**	**OR (CI)**	**OR (CI)**	**OR (CI)**
Any ADL Impairment (ref = No)	6.6 *** (4.0 – 10.9)	5.3 *** (3.1 -8.9)	6.3 *** (3.8 – 10.4)	19.5 ** (2.7 – 142.2)
Pneumonia (ref = No)	3.6 *** (2.1 – 6.0)	2.9 *** (1.7 – 5.0)	3.7 *** (2.2 – 6.2)	4.1 *** (2.5 – 6.9)
AMI (ref = No)	9.0 *** (3.1 – 26.3)	13.1 *** (4.5 – 37.8)	11.8 *** (4.0 – 34.4)	9.9 *** (3.5 – 28.3 )
Sepsis (Ref = No)	5.8 ** (1.9 – 17.5)	5.5 ** (1.8 – 16.4)	5.3 ** (1.7 – 16.1)	6.6 ** (2.2 – 19.3)
Renal Failure (ref = No)	6.1 ** (2.0 – 18.7)	6.4 ** (2.1 – 19.7)	7.0 ** (2.3 – 21.4)	5.7 ** (1.9 – 17.1)
CCI ≥ 3 (ref = CCI < 3)	2.1 ** (1.3 – 3.4)	1.9 ** (1.2 – 3.1)	2.1 ** (1.3 – 3.3)	2.2 ** (1.4 – 3.6)
Chinese (ref = No)	-	2.1 (1.0 – 4.6)	2.2 (1.0 – 4.8)	2.1 (1.0 – 4.6)
Age	-	-	-	1.0 * (1.0 – 1.1)
c-statistic	0.83	0.82	0.84	0.81
AIC statistics	521.95	535.78	522.27	547.56

*P < 0.05 **P < 0.01 ***P < 0.001.

ADL: Activities of Daily Living; OR: Adjusted Odds Ratio; CI: 95% Confidence Interval; AMI: Acute Myocardial Infarction; AIC: Akaike’s Information Criterion; CCI: Charlson Comorbidity Index; ref: Reference group.

Before the inclusion of any functional status measurement, the c-statistics of the baseline models ranged between 0.75 and 0.77. After the incorporation of functional status, the discriminatory power of each of the four models improved. Model discrimination improved the most when pre-admission (from 0.75 to 0.83) and on-admission (from 0.76 to 0.84) ADL impairment based on interview were added.

The pre-admission models using interview data predicted mortality better than the model using medical record. Similar results were found for models incorporating on-admission functional status. However, the difference in the discriminatory power was not statistically significant. Overall, when we compared the models using AIC scores, the model incorporating pre-admission interview data performed better because it is more parsimonious. The *P* value of the Hosmer-Lemeshow statistic ranged between 0.34 and 0.81 across the four models, indicating a good fit for the original data set.

## Discussion

The results of the study indicated that the medical record is a good source of information on functional status prior to admission. The level of agreement in the difficulty experienced by the patient in performing at least 1 of 7 basic care skills (feeding, bathing, grooming, dressing, toileting, transferring and walking) was found to be higher than that reported by Bogardus *et al.*[6]. (к = 0.64 versus 0.48). Variations in institutional emphasis on documentation could have influenced the difference in the extent of missing documentation, and in turn, the level of agreement between the two data sources. The inclusion of specific functional status checkboxes for history taking in the medical records at the study hospital resulted in missing documentation of only 1.1%, compared to 9% reported by Bogardus *et al.*[6].

In contrast to pre-admission information, the extent of concordance between interview and documented impairment in at least 1 of the 7 ADLs on-admission was low (к = 0.28). While other authors [6,15] have similarly found health care professionals to not document functional status accurately, most highlighted the tendency to under-document. However, our study found the prevalence of on-admission ADL impairment to be systemically higher when medical records were used.

Potentially, patients could have under-reported their on-admission functional deficits [30] or health care professionals may have overstated the degree of assistance required. As the share of patients requiring assistance in at least 1 of the 7 ADLs recorded in the prospective interview (50%) is similar to rates reported in studies conducted on comparable patient populations [7,31], the likelihood of over-documentation in the medical records is higher. Contextual factors may have contributed to this. In our local setting, the strong emphasis on falls prevention in the hospital may have created a cautious atmosphere with regards to allowing physically activity in the wards. Patients may be observed and documented to require assistance in bathing, toileting, transferring or walking even though self-report may indicate otherwise. Secondly, with an average bed occupancy rate of 90% and above at our study site, staff are afforded little time in the conduct of a precise functional assessment at admission. Hence, although functional status data were systematically documented through the use of checklist, it is still insufficient to help ensure good data quality.

In addition, across all ADL categories, the agreement between the two sources of data was consistently higher for proxy rather than patient respondents. We postulate that data in the medical records reflected the functional history obtained more frequently from proxies than patients themselves, which is likely for an acutely ill elderly population. This could have contributed to a higher level of concordance between proxy response and medical records.

Several studies have examined and ascertained the importance of functional measures in predicting hospital mortality [4,7,11]. Similarly, our study found that in addition to comorbid disease burden and specific diagnoses of acute myocardial infarction, sepsis, pneumonia and renal failure, the discriminatory power of the hospital mortality model is higher when functional status information was incorporated. Although Covinsky *et al.*[12]. found that ADL impairment prior to admission is a stronger predictor of survival at 1-year than ADL function on admission, we found that the lack of functional reserve in an elderly person before hospitalization predicts survival probability as well as function on admission. One possible reason was that many subjects who died were functionally impaired both before and at hospitalization. In fact, both pre-admission and on-admission functional status are strongly correlated with each other, given that the latter is a composite of the former and the effect of acute illnesses.

Regardless of the data source from which functional status information was derived, the performance of the mortality prediction model did not differ in a statistically significant manner. This is expected when comparing models incorporating pre-admission functional status due to the high level of agreement between the two sources pertaining to impairment in transferring. Although the agreement between on-admission data from the two sources was poor, we found that documented and reported impairment in transferring still sufficiently discriminated between patients with poor prognosis and those who are likely to be discharged alive. A higher share of patients requiring assistance in transferring died in hospital compared to those who are functionally independent.

In interpreting our findings, it is important to consider several limitations. Firstly, as this is a single site study, the generalizability of the results may be restricted. This applies in particular to the agreement between the medical records and patients’ self-report as both data sources might be influenced by institutional norms prevalent in different settings. Nevertheless, our finding of the importance of functional status information in predicting mortality is likely to be generalizable to other settings. Secondly, we did not consider the impact of the patients’ severity of illness on the prediction of hospital mortality. However, as function reflects the impact of the illness on the whole person beyond the extent of organ system derangement or physiologic decompensation [4], it could also account for part of the variability in mortality due to severity of illness.

For researchers, the accuracy of medical records pertaining to patients’ functional status varies depending on whether pre-admission or on-admission information is required. Our results suggest that for studies utilising pre-admission ADL impairment, the medical records is a good source but caution needs to be exercised for on-admission information due to systematic misclassification, which may create subsequent problems in interpreting the parameter estimates when it is used as a candidate predictor.

For policymakers, our study confirmed the need for an accurate assessment and documentation of patients’ functional status. Other authors [6] advocated the augmentation of medical records with functional assessment during nursing admission assessment. As pointed out by Covinsky *et al.*[12], such data may still not be complete. Our findings point out that in a busy hospital environment, beyond functional ability reported by patients, documented data can also reflect care practices. Therefore, even with systematic collection of physical status information, understanding of the primary purpose for the data collection is pertinent in the assessment of its suitability as a risk-adjustor or predictor for evaluating the quality of care and health-related outcomes.

## Conclusion

We conclude that the medical records can be a good source for pre-admission functional status measure but on-admission information was not documented accurately. The discriminatory power of the hospital mortality prediction model was significantly improved with the incorporation of functional status information but it was not affected by their time reference or source of data.

## Competing interests

The authors declare that they have no competing interests.

## Authors’ contributions

WST conceived of the study, participated in design of the study, performed the statistical analysis and drafted the manuscript. YYD supervised the study design, participated in analysis of data, assisted in drafting and editing of the manuscript. WFC, JCT, and JYT participated in study design, drafting and editing of the manuscript. All authors read and approved the final manuscript.

## Pre-publication history

The pre-publication history for this paper can be accessed here:

http://www.biomedcentral.com/1472-6963/12/115/prepub

## Supplementary Material

Additional file 1 Detailed comparison of interview and medical record data for basic ADL impairments by respondent type (N = 1402). Click here for file
